# “Personal recovery depends on NA unity”: an exploratory study on recovery-supportive elements in Narcotics Anonymous Flanders

**DOI:** 10.1186/s13011-020-00296-0

**Published:** 2020-07-31

**Authors:** Anne Dekkers, Sam Vos, Wouter Vanderplasschen

**Affiliations:** 1 Department of Special Needs Education, Ghent University, Henri Dunantlaan 2, 9000 Ghent, Belgium; 2 Yes We Can Clinics, Groenendaal 1, 5081 AM Hilvarenbeek, The Netherlands

**Keywords:** Recovery, Addiction, Mutual aid organizations, Narcotics Anonymous, Qualitative research, CHIME-D

## Abstract

**Background:**

Mutual aid organizations, such as Narcotics Anonymous (NA), can provide support in substance use disorder (SUD) recovery processes. However, research on NA and its recovery-supportive elements is scarce and perspectives of NA-members remain understudied, in particular outside the US. Therefore, this study aims to gain insight into recovery-supportive elements of NA, as experienced by its members.

**Methods:**

To explore the perspectives on and experiences with recovery-supportive elements in NA, 11 in-depth interviews with NA-members were conducted in Flanders (Belgium). Interviews were audio-taped, transcribed verbatim and analyzed by using CHIME-D, a personal recovery framework (Connectedness, Hope, Identity, Meaning in life, Empowerment, Difficulties) developed by Leamy and colleagues in 2011.

**Results:**

Various recovery-supportive elements of NA were highlighted, with Connectedness as a key component including opportunities for building up a social network and for providing a safety net or sounding board. Elements that enabled Connectedness were 1) a non-judgemental approach, and 2) mutual understanding through sharing in NA. Other elements of the CHIME-D framework were less frequently mentioned, although these were inextricably linked to Connectedness.

**Conclusions:**

Connectedness appeared to be the crucial recovery-supportive element in NA, emphasizing the relational character of SUD recovery. Although other elements of the CHIME-D framework were identified, these were closely related to and intertwined with the concept of connectedness.

## Background

Processes of substance use disorder (SUD) recovery are found to be unique and their idiosyncratic nature is acknowledged [1, 2]. Consequently, there is great variety in possible recovery pathways and a range of support and treatment options need to be available to support recovery [3–6]. The intervention spectrum includes, besides formal treatment and support services, also non-professionally assisted programs (e.g., mutual aid organizations) to support recovery processes [7–13]. Recent research by Kelly and colleagues [6] has shown that such informal support groups are as important as formal treatment options to achieve recovery. In their sample of persons who resolved an alcohol and drug problem by assisted means (53.9%, versus 46.1% in unassisted recovery), mutual-help support was the most frequently utilized form of support (45.1%).

Mutual aid organizations are diverse in type and design and 12-step programs such as Alcoholics Anonymous (AA) and Narcotics Anonymous (NA) are probably the most commonly known and largest organizations [11, 14]. AA was founded in 1935, implementing the 12 steps as an instrument for sober living and gradually developed its organizational principles in 12 traditions as it expanded over the years. The AA-program – with its specific focus on alcohol – was adjusted to provide support for individuals struggling with addictive substances, leading to the emergence of NA in 1953 [15]. The NA-program focuses – through NA-meetings and the implementation of NA’s 12 steps in daily life – on personal growth, reduction of egocentrism, supporting others in their recovery journey and a life guided by spiritual principles [11, 13, 16–18]. Whilst the NA philosophy was rooted in the disease model – including the belief in total abstinence – it also recognizes that a radical change in life(style) is required and abstinence in itself is not sufficient to achieve and maintain recovery [13, 16]. The latter aligns with the ‘personal recovery’ paradigm, in which the idiosyncratic, dynamic, process-oriented and multidimensional nature of recovery is endorsed [1, 2, 19–21]. In their search for essential elements for promoting personal recovery, Leamy and colleagues [22] performed a systematic review of the mental health literature, resulting in the CHIME framework. CHIME refers to five recovery supportive elements: Connectedness (e.g., belonging, peer support and relationships), Hope and optimism (entailing dreams, aspirations, and motivation), Identity (comprising a positive sense of identity), Meaning (covering meaning in life and spirituality), and Empowerment (including control over life and focus on strengths) [22]. Recently, the CHIME framework was expanded by Stuart and colleagues [23] who added an extra theme, ‘Difficulties’, including obstacles and challenges in recovery processes. The CHIME-D framework does not only seem suitable to support mental health recovery, but can also be applied to gain insight in and strengthen recovery supportive elements in SUD treatment and mutual aid groups [24–26].

Available studies on recovery and mutual aid have often focused on peer-delivered or 12-step programs (in particular on AA), indicating that these programs facilitate recovery processes by providing specific mechanisms of change (i.e., cognitive, social, affective and spiritual) [27]. A recent Cochrane review demonstrated the efficacy of AA participation for achieving abstinence [28]. Primarily uncontrolled, longitudinal studies indicate that, for example, the presence of and support from peers and sponsors (i.e., peers who have more experience in NA than the peers they support [sponsees]) – often resulting in hope and connectedness – facilitates change [29–34]. Spirituality has been found to be a key element for tackling problems [29, 34]. As a result, participation in mutual aid programs can decrease substance use or induce abstinence [9–11, 29, 35, 36], and can also enhance psychosocial functioning [36]. Furthermore, participation in mutual aid programs reduces health care expenses by its non-professionally assisted approach [11, 28].

Relatively little research has focused specifically on NA [37, 38] and its recovery-supportive elements. Available quantitative evidence on NA indicates that long-term involvement in NA-meetings is positively related to psychological well-being [38]. Moreover, regular attendance at NA-meetings and implementing the 12 steps can enhance social support and reduce alcohol use [39], while commitment to NA-peers and perceived spiritual awakening in NA contribute to decreased craving [40]. Qualitative research that sheds light on personal experiences with NA-groups is limited and shows that recovery processes in NA are supported by personal and psychological factors (e.g., being part of NA, insight in substance use disorders), as well as social features (e.g., transforming social networks, reclaiming roles in society) [41], resulting in improved quality of life [42]. Recovery in NA entails developmental and transformative processes, supported by the available structure of the program (i.e., meetings and 12 steps), spirituality and the undeniable role of peers in sharing experiences and providing hope [43–45].

Given the limited qualitative, in-depth research conducted in NA, few information is available with regard to the personal perspectives and experiences of members on recovery-supportive elements of NA. Therefore, this exploratory study will focus on the recovery experiences of Flemish NA-members and their perspectives on NA and its recovery-supportive elements. To provide suitable and effective SUD recovery support, insight into what is experienced as recovery-supportive and how this works is valuable. This knowledge can be applied to promote participation in NA (or other mutual aid programs) and, possibly, to implement recovery-supportive elements of NA in formal support and treatment programs.

## Methods

### Setting and participants

To explore the perspectives of Flemish NA-members, in-depth interviews were conducted between January and April 2018 with 11 persons in SUD recovery. Whilst NA has over 70,000 weekly meetings worldwide [46], NA was only recently implemented in Flanders (the Dutch-speaking part of Belgium) and is relatively small with around 30 weekly meetings, compared to over 300 AA-groups [47, 48]. After consent of the Flemish area of NA Belgium, information was posted on the website and members were informed about the study during meetings (by the contact person). Eligibility criteria were: 1) being over 18 years; 2) being in self-defined recovery, 3) having received NA-support during the recovery process, and 4) mastering the Dutch language well enough to be able to take part in an interview. Interested NA-members contacted the first author by e-mail or phone and individual interview appointments were made. An overview of participant characteristics can be found in Table 1.

**Table 1:** Participant characteristics

Gender	Male	8
	Female	3
Age	Range	22–51 years
	Mean	37 years
Daily activities	Employed	8
	Student	3
Main substance	Alcohol	3
	Speed	1
	Cocaine	3
	Heroin	1
	Poly use	3
Time in recovery	Range	4 months to 25 years
Marital status	Divorced	2
	Married	4
	Unmarried	5
Children	Persons with children	6

This table presents the participant characteristics of the sample included in this study.

### Data collection

This study was approved by the Research Ethics Committee at Ghent University and all participants provided informed consent prior to their interviews. All interviews started with identifying previous substance use to get some background information on this period in the lives of the respondents. This enabled us to discuss transitions towards recovery. Perceived supportive and hindering factors in recovery were explored and the specific role of NA in their recovery process was discussed. These semi-structured qualitative interviews provided an opportunity for respondents to express their experiences, emotions and perspectives, without being limited by fixed answering categories [49]. Though the researcher used an interview format including the afore-mentioned topics, the story of each respondent was put central and the format was only used to cover some core topics such as general recovery experiences, supportive or hindering elements in the recovery journey and experiences with NA. Interviews lasted between 70 and 100 min. At the end of the interview, respondents received a 15 euro supermarket gift card.

### Data-analysis

All interviews were audio-recorded and transcribed verbatim. All transcripts were read several times to get immersed in the data. During this process it became apparent that Connectedness stood out as a recovery-supportive element and was related to, impacted by and impacted on other supportive elements, which corresponded well with the CHIME-D framework for personal recovery among mental health populations [22, 23]. Therefore, this framework [22, 23] was used to analyse the data. Transcripts were coded line by line based on the master themes (i.e., CHIME-D) and subthemes were added using thematic analysis [50]. Master and subthemes were put in separate tables for each interview. After constructing this codebook per individual interview, master and subthemes from all interviews were brought together to gain insight into and structure the main recurring themes as structured by the CHIME-D framework. The codebook was then extended with quotes related to master and subthemes to have a reliability check between the codes and original data. Throughout the process of data analysis, the first author (AD) discussed and reflected upon the preliminary findings with the second author (SV). The latter was included in this study as a co-researcher with extensive knowledge and lived experience in NA. Involvement of this co-researcher (SV) enabled in-depth and thorough analyses of the data, by combining ‘outsider’ and ‘insider’ perspectives [51]. Throughout the data analysis, it appeared that Connectedness was by far the most frequently mentioned master theme in coding the data. Therefore, this theme will be the starting point of the results section presented below. Additionally, Connectedness is used to discuss other elements of the CHIME-D framework as it appeared that Hope, Identity, Meaning in life and Empowerment are almost inseparably linked to this theme. Difficulties were hardly mentioned as stand-alone items, but were mentioned in relation to various components of the CHIME framework.

## Results

During data analysis two core elements came to the surface, related to respectively the ‘what’ and ‘how’ of recovery support within NA: (1) Connectedness as a key factor for supporting personal recovery*;* and (2) NA features facilitating Connectedness. Also, elements of Hope, Identity, Meaning in life and Empowerment that are closely related to and associated with connectedness are discussed. Given the undeniable interrelatedness of these themes, these elements are presented as intertwined with Connectedness, since a separate presentation would contradict their mutual relation.

### Connectedness as a key factor for supporting personal recovery

*‘I go every week [*to an NA-meeting*] and that gives me – because I am fairly new there – a sense of belonging, and I hope I give something back.’* (Male, 40s).

Respondents mentioned that Connectedness is the main impetus for recovery and is provided in NA-meetings and by fellows. Most respondents mentioned *togetherness* as an important asset. This means relationships are built towards friendships and for some even towards ‘family-like’ bonds. Consequently, respondents no longer felt alone. Under the common denominator of recovery, people who usually do not necessarily cross paths came together in NA and found connection with and support from each other. Rudi mentioned he finally feels like he is ‘at home’ in NA:

*'Recently, I was at an NA Christmas party […*]. *You sit at the table with strangers and you just sit there chatting about feelings, emotions, about things that you have experienced that were difficult for you. […*] *I thought that was really great. I feel at home there. When I go to a meeting like that I feel at home […*]. *Then I can say: “I’m Rudi, I’m an addict” and that’s okay'.* (Male, 20s).

In order to build and contain this feeling of Connectedness, four respondents mentioned that it is crucial to frequently attend NA-meetings. By doing so, bonds with other members and the NA-program were perpetuated. Some mentioned that when cutting down on meeting attendance, for example when life and recovery go seemingly well, they felt Connectedness began to dilute which often resulted in a setback in the recovery process. *Staying connected* therefore seemed vital. Ruby compares it to a yearly car maintenance:

*‘It’s pretty much like having my yearly car maintenance. It makes my car drive better, longer, further [ …*]. *If I don’t have the maintenance done – ok it [*the maintenance] *does cost me 200 Euros – and then something breaks down, it is usually something serious. And actually, NA-meetings are just some sort of weekly maintenance. I come with my ‘car’ and my ‘car’ drives better. And yes, it will not immediately break down if I do not go into ‘maintenance’, but you must be aware of what you are doing then.' *(Female, 40s).

As mentioned by seven respondents, attending NA-meetings enabled them to *build a social network*. For some, this NA-network is complementary to an existing social network not related to substance use or recovery (e.g., non-using friends). For others, the NA-network is filling the gap that is left by abandoning their old ‘user’ network. Whilst respondents mentioned the need for an extended recovery-supportive network (i.e., a network that is broader than NA-peers), the NA-network can be a starting point from which NA-members can socialize (again) and build new relationships.

Some respondents stated that the NA-peer group – in contrast to, for example, peer groups in a therapeutic community – is continuously supplemented with new members. As a result, there is always a group of peers available to connect with and provide hope (Hope). Rudi suggests this allows to ‘stick with the winners’:

*‘I attended my first meeting and there were two people who were in the program for 20 and even 30 years. That provided hope. In a TC program, you see people leave and come back after 3 weeks and who relapse over and over again. In NA, this also happens, but less. They say ‘Stick with the winners’ and when I see them* [the winners], *that really means hope for me.’* (Male, 20s).

Eight respondents mentioned the group is experienced as a safety net or a sounding board. This safety net means having a network that understands their situation, challenges, and that is available, also outside meetings, when recovery is hampered by, for example, craving. Some respondents referred to the group as a sounding board that offers – through confrontation and connection – new perspectives. Also, when they are doing well, a brief conversation with NA-peers may be needed to confirm this status.

Spirituality has an important recovery-supportive role in NA according to nine respondents, for instance in finding a ‘power greater than yourself’ (Meaning in life). NA does not determine in advance what this power is or can be, but NA-members are encouraged to discover what this power could be for them (e.g., the NA-group, meditation, a god). This ‘higher’ power provides support and can function as something to rely on.

### NA features facilitating connectedness

Since unconditional Connectedness is a key factor in NA, we further elaborate on two features that enable Connectedness according to respondents: 1) a place free of judgement; and 2) sharing and listening to experiences, resulting in mutual understanding and the creation of a new identity.

#### A place free of judgement

Several respondents pointed out they particularly appreciate *the withholding of judgement* in NA-relationships and meetings. A non-judgemental approach is stated to be beneficial on both ends of the scale for NA-members: when sharing their own story, yet also when listening to other NA-members sharing their story. When sharing their story, NA-members felt safe, understood and accepted, since no judgement was given in the absence of cross-talk. As Tess (female, 20s) mentioned, being able to share your story – maybe even multiple times – provides a chance to gain insight in your own story without the interference and judgement of others (Empowerment). This non-judgemental and safe space enhances motivation for change and provides opportunities for growth.

*‘I think that is interesting because you can hear yourself talk, literally, all the time. [ …*] *You need to hear yourself talk and sometimes people share the same thing five times, because they are stuck in it. And the 6*^*th*^*time, they feel like: “What am I actually saying?”. You see, they suddenly understand, they suddenly hear their own story and they feel like: “Ah yes ok, I understand. I know what to do*” *. That is the thing and actually you do it together, but still you need to grow from the inside.’* (Female, 20s).

On the other hand, respondents state they learn from listening to others who share their (mostly recognizable) stories without the need to interfere with these stories. By avoiding cross-talk, NA-members learn to move away from judgement and (unwanted) advice, and learn how to listen to others.

#### Sharing experiences resulting in mutual understanding

Related to the above, sharing – by giving and receiving – with NA-peers was perceived to be of vital importance to experience mutual understanding.

##### Finding meaning through ‘giving’

*Giving* can entail the mentioned opportunity to share personal stories with NA-peers. As a result of the safe context in NA, some respondents share a lot more with NA-peers compared to what they share with families and friends.

*‘I share more at meetings or with fellows than with my brother or my parents. Yes, my brother knows most about me at least outside of NA, but NA knows everything.’* (Male, 20s).

By sharing with NA-peers, it became possible to vent feelings and to provide openness for discussion with regard to difficult situations. Furthermore, five respondents stated that by sharing their story, they appreciated they could inspire others and give hope to others that recovery is possible (Hope). Also, by sharing their story with others, they did no longer have to bear it all by themselves:

*‘You tell someone and then it is out there. You then no longer have to bear it completely on your own.’* (Female, 20s).

The power of giving goes beyond sharing personal stories, it can also include finding satisfaction in giving back to NA or NA-peers. Ruby (female, 40s) does so by supporting the continuation of NA and being continuously present at NA(-meetings), as she noticed the need for participation of NA-peers with considerable ‘clean time’ (in her case, 18 years). Being able to provide support for NA-peers is found to be important and may include being a sponsor for a fellow. For five others, giving means ‘serving’ in NA (i.e., being responsible for a specific element of NA(-meetings) such as a coffee-person). Through this ‘service’ respondents embrace new roles that are meaningful to them (Meaning in life) which provide a satisfying connection (Connectedness) and a sense of responsibility (Empowerment).

*‘In NA, I do service. I have three different roles: I am responsible for the literature, I am the coffee person and I am responsible for the events throughout Flanders. Then, you try to give something back to NA.’* (Female, 30s).

*‘Friday evening I do service, I am the treasurer. It is some kind of obligation. I go every week and that gives me, because I am fairly new here, some sense of belonging and I hope I can give something back. I pay the rent, count the money, pay the coffee, the literature. It is not a difficult task, but it is an important one [ …].I am really consistent and it affects me if I cannot do this service, then I crawl back into my shell.’* (Male, 40s).

Moreover, being supported by NA-peers to take on a specific ‘service’ can enhance self-confidence (Identity).

*‘Because of these NA social events, I was trusted by the people because they saw that I was doing very well and I was still coming to meetings after those 90 days. [ …]So that means having confidence and you get it.’* (Male, 30s).

Six respondents mentioned taking on new roles is mostly accompanied by changes in their daily routines (Meaning in life). Respondents stated that by attending NA-meetings, by working on their 12 steps and by connecting with NA-peers, they developed new routines. As respondents redesigned their lives, their weekly schedule was often affected by NA-membership with meetings taking a substantial part of their time allocation. Furthermore, they often spent leisure time with NA-peers.

*‘It [attending NA meetings] has become a structural part of my life. I never questioned that I will continue this for the rest of my life. I will never use again and I will never stop attending NA meetings.’* (Male, 40s).

##### Building on others

Respondents highlighted that besides giving, they also *receive* from NA and NA-peers. For example, support from a sponsor during recovery can be extremely helpful, since more experienced NA-peers can provide suitable and tailor-made support based on shared experiences and Connectedness and attuned to the needs of sponsees (e.g., support in the event of breaking up a relationship, practicing how to communicate about emotions). Philip (male, 30s) mentioned his sponsor had a very specific approach that focused on ‘ruining’ his relationship with substance use:

*‘He told me from the beginning, I’m not going to help your recovery, I’m going to screw up your use. And that’s pretty cool, because if you look at it that way: he is not going to help me with my recovery, but there are certain things that I say, and he just repeats them, just says it out loud to me. And then you will hear it* [what you say] *from someone else. [ …]So he’s never going to tell me what to do.’* (Male, 30s).

To *receive* further included what NA-participants learn from peers and during meetings. By listening, without judgement, to stories from peers, respondents gained insight into their own change processes. Not only stories of experienced NA-members were valued, but also stories of those new to NA and recovery were equally important. Although stories of experienced and new NA-members clearly differ, they are both seen as relevant and complementary.

To install Hope and motivation for recovery, respondents can call upon a wide range of NA-peers. Hope arises on the one hand by observing and listening to those who are in long-term recovery, as the idea that stable recovery is possible enables trust. On the other hand, newcomers are a constant reminder of the downsides of substance use. They counterbalance the sometimes diminishing awareness of the negative experiences with substance use for those who have been in recovery for some time.

Six respondents mentioned their self-efficacy (Empowerment) grew by attending NA-meetings. Moreover, some expressed that the NA-program facilitated time to reflect about the question ‘who am I?’ (Identity). Getting to know themselves offered the opportunity to accept themselves for who they are, yet also to take the challenge to further develop a new identity throughout the recovery process. Connectedness enables the construction of a new identity as a person in recovery (Identity). Eight respondents felt empowered in dealing with emotions and difficulties (Empowerment) through peer contacts, as Edgar (male, 50s) mentioned:

*‘I used to be not so open* [in communication] *and now I am much more open and I am able to listen and get suggestions and gain insight. I used to think I was always right and that I knew it all. I was going to do it all myself, but addiction cannot be addressed solely by yourself, you cannot. That is why the NA group is so important, because we are together to address it together.’* (Male, 50s).

##### Mutual understanding as building block for Connectedness

Being able to feel connected and to give and receive from NA-peers eventually results in *mutual understanding*. Half of the respondents explicitly mentioned that Connectedness grows by recognizing yourself in NA-peers. The identification with stories from NA-peers enhanced mutual understanding. Furthermore, knowing and understanding what peers have been through, enabled them to support and confront each other and limited the options to ‘keep up appearances’. With SUD and recovery as the common denominators, peers connect with each other as Tess (female, 20s) puts it:

*‘I don’t know, you feel less weird there, because you hear people talk about themselves and you hear yourself constantly. I think it is very recognizable. You feel like: “Ah I have that too, I am exactly like that”. You feel less weird, I think. [ …*] *I don’t know it is a kind of unity or something. That you feel like, okay you know, maybe I am weird, but we are all the same and that is ok.’* (Female, 20s).

## Discussion

With this study we aimed to explore personal recovery among Flemish NA-participants and recovery-supportive elements broader than abstinence in NA groups in Flanders, using the CHIME-D recovery framework [22, 23]. Based on 11 interviews, various recovery-supportive elements were found in NA, with Connectedness as a central component. Though we focused almost unilaterally on specific recovery-supportive elements of NA, it must be noted that recovery support goes beyond the setting of NA. NA provides mutual help based on regular meetings in the community, supporting recovery processes of NA-members that are grounded in individuals’ ‘natural’ personal, social and societal contexts [13, 16, 20, 52]. As a result, there is a continuous interplay between these settings and related recovery-supportive (or impeding) elements [20, 53]. Keeping this in mind, we focus here on the central role of Connectedness and the relational nature of personal SUD recovery in NA. In addition, we reflect on the complexity and multidimensionality of SUD recovery processes and how this relates to the application of the CHIME-D framework [22, 23].

### Connectedness as foundation for change

In this exploratory study, Connectedness (e.g., with peers, sponsors, sponsees) emerged as the primary recovery-supportive element in NA, complementing the current personal recovery paradigm [1, 2, 20, 21, 54]. Meeting others in a place that is free of judgement, where they can build mutual understanding through sharing their perspectives and learning from others’ experiences was mentioned as crucial for initiating and maintaining recovery [1, 19, 55, 56]. In NA, members have continuous options for Connectedness, even when individuals retreat from participation (e.g., in the case of relapse). Mudry and colleagues [57] have underscored the importance of ‘healing interpersonal patterns’ to substitute ‘pathologizing interpersonal patterns’ that were present during active substance use. In NA, numerous healing interpersonal patterns are installed through mutual understanding and Connectedness, creating an atmosphere that facilitates recovery through connections with others [43, 44, 57]. However, whilst strong bonds in recovery-supportive groups can enhance recovery, they may induce a feeling of ‘us’ and ‘them’, resulting in barriers towards exploring and utilizing support outside the own group [58]. Consequently, several challenges remain to bridge the gap between the recovery-supportive environment of NA and the wider community where NA-members live and work.

To build Connectedness and support recovery Abedi and colleagues [43] and the present study emphasize that ‘experienced’ NA members as well as ‘newcomers’ are important (i.e., ‘experienced’ peers provide hope that stable recovery is possible, ‘newcomers’ provide hope by sharing their initial recovery steps and also confront members with the negative effects of substance use). Interestingly, this finding is in sharp contrast with the study by Snyder and Fessler [59] who indicated that despite the egalitarian principles of NA serious status differences may emerge between more ‘experienced’ members and ‘newcomers’. These different findings might be explained by sampling differences (e.g., ‘enthusiasts’ in the present study) and potential barriers NA-members experience in raising difficulties they encounter in NA as put forward by Christensen [60]. Therefore, a comprehensive approach is warranted when studying experiences with NA, in order to capture essential supportive components but also experienced difficulties from the perspectives of a diversity of respondents (e.g., enthusiasts, ‘drop outs’).

Connectedness emerged as a central supportive element in NA throughout this study. The importance of Connectedness has been established in previous studies in mental health as well as SUD recovery research. For example, the recent study by Mudry and colleagues [57] suggests that strong Connectedness between professionals and service users promotes change in recovery processes. The current study, however, contributes to specific insights on Connectedness within NA and the role NA can have in supporting recovery in Flanders. The organization of SUD treatment and support in Belgium and other European countries is - as opposed to, for example, the United States – not merely based on nor grounded in 12-step facilitation programs [61, 62]. SUD treatment in Belgium entails a wide range of services, from universal prevention to long-term treatment in drug-free therapeutic communities, also including outpatient counselling centers, harm reduction services and diverse hybrid (not 12-step based) residential programs [63]. Consequently, NA (and AA) are not closely related to nor part of the formal treatment system and, therefore, referrals to NA as an alternative to professional treatment or as a type of continuing care are less common in Belgium. Enhanced participation in NA can be stimulated by informing professional SUD workers on NA and mutual aid in general, since previous research [64–68] demonstrated that referral to mutual help groups may be impeded by their negative perceptions or limited knowledge on mutual aid groups. As Best and colleagues [64] found, providing training for professionals about mutual aid groups and its role in stimulating Connectedness can increase professionals’ understanding and expand the referral options for the persons they work with. Additional research in Belgium and other countries where NA is not widely implemented could focus on the perceptions of professionals who work with persons with SUDs and the need to make professionals more acquainted with peer-based support and the important role these groups can have in a recovery-oriented system of care [69]. By raising awareness amongst professionals, these groups become more visible and accessible for those seeking support and for their social network looking for adequate support for their loved ones [64, 70].

Furthermore, additional research on the supportive features that emerged from this small-scale study on NA in Flanders – such as Connectedness, non-judgemental approach, mutual understanding and the creation of a new identity – could be designed more rigorously and by combining qualitative and quantitative methods to further explore specific hypotheses. Eventually, this will contribute to an enriched pallet of support options, with mutual aid programs as an integral part of an integrated system of SUD support [69, 71].

In short, NA provides support that enables and reinforces Connectedness, which is, in turn, found to be an essential component of personal recovery processes [22]. Emphasizing the essential role of Connectedness affirms recovery as a unique and personal, yet relational process, illustrating its social nature [41, 52, 57, 72, 73]. Consequently, an interactional perspective on SUD recovery is warranted [52, 57, 72]. Recovery goes beyond individuals’ responsibilities and the necessity of connections with and support from a social network and society at large is widely recognized (e.g., the importance of social and community recovery capital) [2, 20, 72, 74, 75]. Understanding recovery as a relational and social process is essential to provide adequate recovery support [52, 73].

### CHIME-D: artificial categories or a holistic approach?

By analyzing the data using the CHIME-D framework – originally developed in the field of mental health recovery, but also found applicable in the field of SUD [22, 24, 25] – Connectedness was identified as the pivotal supportive element for SUD recovery in NA. As recovery is a relational process [52, 57, 72, 73], changes in Identity, Hope, Meaning in life, and Empowerment can only take place in relation to others (Connectedness). For example, by embracing a new meaningful role by providing services in NA (Meaning in life) or rebuilding identity through connections with and mutual understanding between NA-members (Identity). Change occurs during encounters with others in a multitude of relationships [73]. While Connectedness has been identified as the key factor for personal recovery, it does not unilaterally impact on Hope, Identity, Meaning and Empowerment as the development of these elements tends to affect – in turn – on (strengthening) the sense of Connectedness. Consequently, based on this study with NA-members in SUD recovery, the CHIME-D elements appear strongly intertwined with and related to each other and a strict division seems artificial. Moreover, the additional category ‘Difficulties’, an extension of the CHIME framework by Stuart and colleagues [23], do not correspond with our exploratory findings as these indicate that difficulties occurred in connection with others and were related to (a lack of) Connectedness, Identity, Meaning in life, Hope, or Empowerment. Instead of a separate category, difficulties emerged within all CHIME categories and should be perceived as such. Additional research could shed further light on the application of the CHIME-D framework in SUD recovery, with particular attention for the undeniable interconnectedness of these categories identified in this explorative study. When doing so, a holistic perspective which takes into account the multiplicity and complexity of human beings should be applied [76].

Notwithstanding the innovative approach of this study, some limitations should be noted. First, the respondents were mostly NA-enthusiasts. They pointed out the beneficial effects of NA on their recovery process and planned NA-participation in the future. They mentioned very little difficulties with and critical concerns related to NA. Christensen [60] found that NA-members might experience difficulties in questioning the NA-program due to its neoliberal perspective on SUD and recovery. This approach may urge NA-members to focus primarily on their individual responsibility, with little attention for societal and contextual factors related to SUD and recovery. Since we did not elaborate on this topic in the interviews, it remains unclear whether Christensen’s theory [60] applies to the respondents in this study. To broaden the scope on experiences with NA, it is recommended to include not only ‘enthusiasts’ in future research but to involve also individuals with other perceptions on NA. By doing so, the narratives on NA could be extended with perceived difficulties and barriers to recovery support in NA. Second, using a theoretical framework such as CHIME-D [22, 23] has its pitfalls, for example, the risk of indiscriminately applying a model during data analysis. We tried to tackle this by using CHIME-D as the broader framework for our coding tree, yet supplemented with sub-codes that arose through thematic analysis.

## Conclusion

Connectedness was found to be a crucial recovery-supportive element within NA, highlighting the relational character of SUD recovery. NA-specific features such as a non-judgmental approach and mutual understanding through sharing create a climate in which Connectedness can develop. Other CHIME-D elements were mentioned, albeit less frequently and intrinsically linked to Connectedness. It is through Connectedness with others that Hope can emerge, Identity is (re)build, Meaning in life is acquired, and Empowerment is developed. Given the key role of Connectedness, it is important to increase attention for Connectedness in NA Flanders and its role in the wider treatment system. Furthermore, when supporting individuals confronted with SUD and recovery, professionals in Belgium and other countries should be aware of the power of mutual aid organizations, as well as its pitfalls and limitations.

## Data Availability

The data generated and analyzed in this study are not publicly available in order to protect the participants’ anonymity but are available from the corresponding author on reasonable request.

## Abbreviations

NA: Narcotics Anonymous
AA: Alcoholics Anonymous
